# Biodielectrics: old wine in a new bottle?

**DOI:** 10.3389/fbioe.2024.1458668

**Published:** 2024-10-03

**Authors:** Hema Dinesh Barnana, Syed A. M. Tofail, Krittish Roy, Charlie O’Mahony, Veronika Hidaši Turiničová, Maroš Gregor, Ehtsham ul Haq

**Affiliations:** ^1^ Department of Physics and Bernal Institute, University of Limerick, Limerick, Ireland; ^2^ Centre for Nanotechnology and Advanced Materials, Faculty of mathematics, physics and informatics, Comenius University, Bratislava, Slovakia

**Keywords:** Biodielectrics, Biopiezoelectrics, Biopyroelectrics, Bioferroelectrics, low k dielectrics, Pieoelectrics, Pyroelectrics, Ferroelectrics

## Abstract

Biodielectrics is a subset of biological and/or bioinspired materials that has brought a huge transformation in the advancement of medical science, such as localized drug delivery in cancer therapeutics, health monitoring, bone and nerve repair, tissue engineering and use in other nanoelectromechanical systems (NEMS). While biodielectrics has long been used in the field of electrical insulation for over a century, polar dielectric properties of biological building blocks have not been well understood at the fundamental building block level. In this review article, we provide a brief overview of dielectric properties of biological building blocks and its hierarchical organisations to include polar dielectric properties such as piezo, pyro, and ferroelectricity. This review article also discusses recent trends, scope, and potential applications of these dielectrics in science and technology. We highlight electromechanical properties embedded in rationally designed organic assemblies, and the challenges and opportunities inherent in mapping from molecular amino acid building blocks to macroscopic analogs of biological fibers and tissues, in pursuit of sustainable materials for next-generation technologies.

## 1 Introduction

The term biodielectrics can be defined as the materials exhibiting dielectric property which originate in, or derived from, or inspired by living systems in nature. Examples include gutta-percha, wood, amino acids, tissues, bioceramics and bone. They may or may not have undergone further modifications or engineering for the use as dielectric materials. Similarly, they may or may not be bio or environmentally degradable although general expectation will be that they are more readily degradable than similar materials of petrochemical origin such as synthetic polymeric dielectrics. In recent times, biodielectrics has been put forward in many sectors in the field of science and technology, like healthcare (Reibetanz, 2021; Pethig et al., 2003; Grant, 1984), energy harvesting (Venkat et al., 2011), or environment (Arif et al., 2023), as a potential alternatives to replace or substitute conventional dielectric materials. The impetus comes from non-toxicity, biocompatibility, biodegradability, flexibility, sustainability, and relative ease of processing of biodielectrics. As such, and through further engineering of these materials, it may be possible to develop devices for day to day use, for example, as sensors, wearable electronics, and other self-powered devices (Wu et al., 2021; Zaszczyńska et al., 2020; Voit et al., 2015; Knapkiewicz and Kawa, 2023). Alongside, biodielectrics can bring forward advanced medical applications, e.g., in localized drug delivery in cancer therapeutics, real-time health monitoring, bone, muscle and nerve repair, tissue engineering and use in nanoelectromechanical systems (NEMs) (Stroe et al., 2013; Fine et al., 1991; Raghavan et al., 2012; Fernandez-Yague et al., 2015). Due to the inherent and engineerable dielectric properties and environmental sustainability, biodielectrics can become alternative to conventional dielectrics in technical applications including sonars, sensors, accelerometers, resonators, medical devices, wearable electronics, smart devices, and other energy harvesting applications (Singhwal et al., 2021; Pelrine et al., 2001; Kornbluh et al., 2002).

The extensive study of dielectric properties in biological systems started with the discovery of qualitative piezo and pyroelectricity in the wool fibers bounded by shellac in 1941 by A. J. Martin (Martin, 1941). The discovery of piezoelectricity in wood by Bazhenov and Konstantinova in the middle of 20th century and later confirmed by Fukada (Fukada, 1955) developed interests in biological macrostructures. Fukada and Yasuda went on to report shear and tensile piezoelectricity in bone and tendon collagen in 1957 and 1964 respectively (Fukada and Yasuda, 1957; Fukada and Yasuda, 1964). Lang reported pyroelectricity in bone in 1966 (Lang, 1966). Piezoelectricity in amino acid powders was first reported by Vasilescu in 1970 (Vasilescu et al., 1970). Piezoelectric properties in materials of biological origin such as collagen, blood vessel walls, intestines, trachea, horn keratin, DNA, lobster shell apodome chitin, and myosin and actin of muscles became a matter of curiosity, most often qualitatively (Fukada and Hara, 1969; Fukada and Ueda, 1970) (Fukada et al., 1975; Ando and Eiichi, 1976; Ando, Fukada, and Glimcher, 1977; Fukada and Yasuda, 1964; Lang, 1966; Athenstaedt, 1968). Successful discovery and demonstration of piezoelectricity and pyroelectricity of biological materials made researchers look also for a ferroelectric phenomenon in biologicals. Polonsky and Stanford carried out some of the earliest investigations on the bio-ferroelectricity on deoxyribonucleic acid (DNA) (Polonsky et al., 1960) and ribonucleic acid (RNA) (Stanford and Lorey, 1968).

The existence of piezoelectricity in a variety of biological systems and molecules has been established (Stapleton et al., 2016; Karaffová et al., 2021). This led to these structures, which are embedded in almost all living organisms, must play some physiological roles through their endogenous electric fields. Examples include embryo development, tissue regeneration, and neural networks systems (Burr, 1952; Zhao et al., 2006; Levin and Stevenson, 2012; Canadas et al., 2020; Kapat et al., 2020). Piezoelectricity has often been implied as the cause or governing mechanism in callus formation, fracture healing, and tissue and nerve regeneration. Molecular mechanisms of such physiological roles still allude us. The 2021 Nobel Prize in Physiology or Medicine awarded to David Julius and Ardem Patapoutian for their discoveries of temperature and touch receptor Piezo one and Piezo two proteins brings to fore the importance of studying dielectric nature of biological building blocks such as amino acids, peptides, proteins, and tissues. Interestingly, piezoelectric effects have yet to be claimed in the electrical behaviour of these proteins although fibrillar, globular and membrane proteins have all been found quantitatively to be piezoelectric in the classical sense.

**FIGURE 1:**
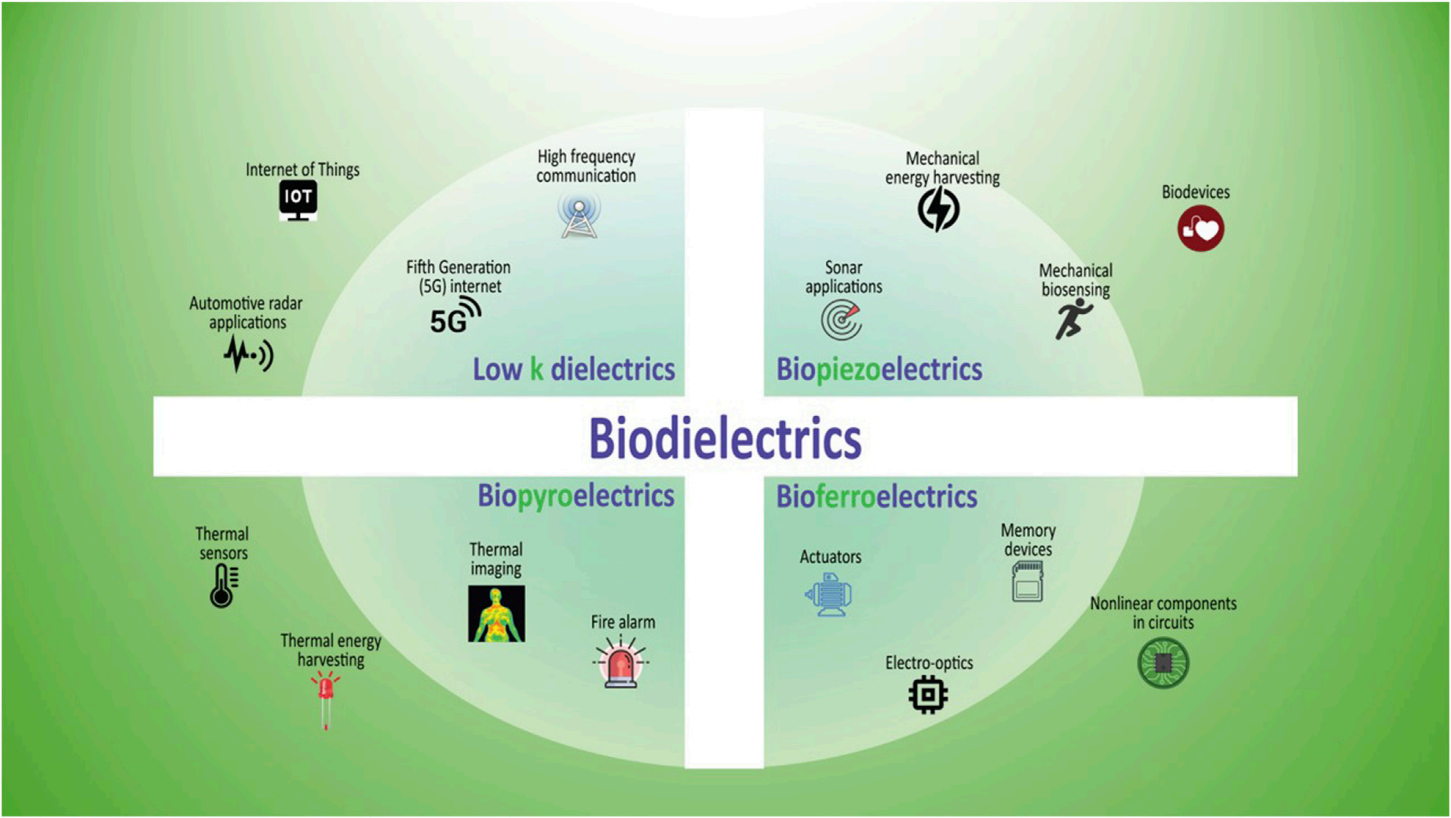
Showing the Biodielectrics that typically belong to the category of low dielectrics, with a more detailed breakdown into Biopieoelectrics, Biopyroelectrics, and Bioferroelectrics, along with their possible utilization in specific fields.

This article gives, after a brief introduction to the fundamentals of dielectrics and their subclassifications, a discussion on biological and bioinspired materials that can be classified as biodielectrics as shown in Figure 1. We discuss recent trends, scopes, and potential applications of the science and technology of these dielectrics in pursuit of sustainable materials for next-generation technologies such as Internet of Things (IoTs), human-machine interfaces, and brain-inspired computing.

## 2 Fundamentals of dielectrics

The term “dielectric” was derived from Greek language ‘‘Dia’’ which means through, and it was first coined by William Whewell in 1837 (Daintith, 2008). A dielectric material is a poor conductor of electricity and is capable of supporting an electrostatic field to store energy and release it upon demand or if necessary. One of the earliest applications of dielectrics is the so-called Leyden jar developed in 1745 (Conway and Conway, 1999). It consisted of a glass used as a capacitor to store charge (Ho et al., 2010; Conway and Conway, 1999). The dielectric undergoes a change in its state (polarisation) when subjected to an external electric field. A few dielectrics retain the stored energy and discharge energy in the form of electrical charge. The ability of a material to store energy storing charges/polarisation in an electric field is shown by all dielectrics. There is another class of dielectrics that can store charges or remain polarized even when the electric field is removed. This class of “electrified” materials are termed as electrets, following the analogy from magnetism that retains magnetization. Most biodielectrics are either natural electrets (bioelectrets) or can be made electrets by subjecting to electrical polarisation.

Based on the alignment of positive and negative charge carriers and the resultant dipole moments, the dielectrics can be classified into two groups-polar dielectrics and non-polar dielectrics. In non-polar dielectrics, the constituting molecules have no net dipole moment because of the match in center of mass of positive and negative atoms. To polarize non-polar dielectrics, we use different poling techniques that are based on the trapping of electric charges. These are, for example, contact poling method, electromagnetic method, electron beam irradiation, corona poling, and thermo-electric method.

On the other hand, polar dielectrics are constituted of molecules with a net dipole moment arising from the non-alignment of center of mass of positive atoms and negative atoms in the molecules. In the thermodynamic equilibrium state the dipoles are randomly oriented and the net dipole moment of a piece of material is zero. However, when subjected to an electric field, the randomly arranged dipole moments in polar dielectrics align in the direction of the applied electric field. Upon removal of electric field, they return to their quasi-permanent level. These materials have held charge for years and used in many aerospace and sonar applications.

Based on the symmetry, 21 of the 32 point groups are non-centrosymmetric meaning that they lack a centre of symmetry and the remaining are centrosymmetric. Centrosymmetric materials show electrostriction, tribo- and flexoelectricity. Non-centrosymmetric dielectric materials can be further divided into piezo-, pyro-, and ferroelectrics based on their response to the applied electric field. Except for the non-centrosymmetric cubic group 432, the remaining 20 non-centrosymmetric groups show piezoelectric behavior (Park et al., 2020). Piezoelectrics is a subgroup of dielectric materials which generates polarisation when mechanical stress or strain is exerted. The converse phenomenon involves an applied electric field which distorts the piezoelectric material causing mechanical strain. These piezoelectric materials are used in applications such as high-voltage generators, gas igniters, positioners, actuators, and transformers.

Pyroelectrics is a subgroup of non-centrosymmetric materials that possesses spontaneous dipoles that allows generating electricity upon application of heat and *vice versa*. Among the 21 non-centrosymmetric group symmetries, 10 groups possess the so called polar symmetry that allow them to show spontaneous polarisation, and exhibit both piezo and pyroelectric properties. Within these 10 groups, a further subgroup possesses reversible spontaneous polarisation. These are ferroelectric materials that exhibit piezo, pyro and ferroelectric properties.

Ferroelectric materials are characteristic by their spontaneous electric polarisation that can be reversed by an external electric field. Usually, there is no iron in ferroelectrics. The name is simply an analogy to ferromagnetic materials, because the properties exhibited by ferroelectric materials, such as hysteresis loop, response to stress, and spontaneous electric polarisation, are analogous to ferromagnetic materials. Few exhibits spontaneous polarisation and forms hysteresis (similarly to ferromagnetic materials which retain the magnetism even after removal of field) and they are called ferroelectrics.

## 3 Low k dielectrics

As it has been stated before, dielectrics are used in a variety of technical applications and have been employed in a number of potential advanced capacitors, actuators, or transducers applications such as nanogenerators (Zhao et al., 2021), wearable health monitors (Su et al., 2021), smart textiles for electricity generation (Chen et al., 2020a). These involve dielectrics materials with a range of relative permittivity (k). Most of the biological materials, in their native and un-engineered state, possess relatively low range of magnitude of dielectric permittivity. This can be advantageous in numerous applications where low-k dielectrics are actively sought after. For example, the search for new materials for integrated circuits has resulted from the unrelenting quest for microprocessors that are quicker and more efficient. The interconnects within integrated circuits became a bottleneck for performance enhancement at the 0.25 μm technology node. In high frequency communication devices, ensuring faster signal transmission with minimal signal loss requires materials with lower relative permittivity and low dielectric losses. These low k dielectric materials have advantages like lower dielectric loss, faster signal transmission compared to traditional dielectric materials made them to use in high frequency microelectronics such as printed circuit boards (PCBs) and antennas, as well as microwave communication components used in, for example, Internet of Things (IoT), Fifth Generation (5G), inter-satellite gesture sensing, communications, and automotive radar applications. thus, interconnection point of view, usage of low k dielectric materials are necessary to limit electronic crosstalk, charge build-up, and signal propagation delay. Low k dielectrics are considered as materials with dielectric constant lower than that of silicon dioxide (k ∼ 3.9). Researchers developed novel materials to lower capacitance and connection resistance in order to overcome this. One such turning point was the switch from aluminum (Al) to copper (Cu). Silicon dioxide which is used for such applications whose relative permittivity ∼4 which is less when compared with existing inorganic dielectric materials, but still, it is high results in high dielectric losses.

There are two ways to reduce the k value, one is to create pores, and the other is to reduce polarisation at the molecular level by replacing polar atoms or groups with with atoms or groups of relatively low polarity such as replacing oxygen (O) in the structure by C-H. Going to the fundamental aspects of polarizability, the materials containing polar components (e.g., Si-O) has higher dielectric constant compared with the materials containing less polar components (e.g., C-C, Si-F, Si-C, C-H). The dipole formation is a result of electronic polarisation (displacement of electrons), distortion polarisation (displacement of ions), or orientation polarisation (displacement of molecules) in an alternating electric field. These phenomena have characteristic dependencies on the frequency of the alternating electric field, giving rise to a change in the real and imaginary part of the dielectric constant between the microwave, ultraviolet, and optical frequency range. This led researchers to investigate materials that inherently possess relatively low dielectric constants based on less polar polymeric materials such as polyimides, polyphenylene oxides, and fluoropolymers. These materials offer lower dielectric constants than that of silicon dioxide (typically between 2.5 and 4) but often suffers from low thermal stability, poor mechanical properties, and higher moisture absorption. Polymer based or other inorganic low k dielectrics cannot withstand the high processing temperature (Singh and Ulrich, 1999). Here are two ways to answer this, one is the development of low k high temperature resistant dielectric materials, and the other is the development of methods to reduce the processing temperature to use currently available low k dielectric materials (Singh and Ulrich, 1999). Also, these materials often originate from petrochemical precursors and create environmental burden and are often toxic. Sustainability of these materials are often a major concern.

The other way to reduce the dielectric constant is using porous dielectric materials or inducing porosity. Silicon dioxide (SiO_2_), due to its process compatibility, good thermal and mechanical properties, has long been used in semiconductor industry as a robust interlayer dielectric. Dielectric constants can further be brought down by introducing porosity in the structure (Xie et al., 2022). For example, has prepared nanoporous SiOF, into which they injected fluorine molecules to create less polar Si-F. Ultra-low k porous SiCOH materials were created to preserve low capacitance as devices scaled further.

Biodielectrics with permittivity in the range suitable for low k dielectric applications are numerous even without introducing porosities. Biological materials such as jute, cotton, coir have historically used as insulating materials in large scale communication cables, for example, submarine cables for transatlantic telegraph cables. Biodielectrics can thus be important materials to be considered as potential alternatives to conventional inorganic and polymer based low k dielectric materials. One more problem with low k dielectrics is the low thermal conductivity that limits heat dissipation especially in high powder density chips. Biodielectrics can be engineered to possess high thermal conductivity with low dielectric permittivity to address these problems. Inspiration for such engineering strategy can be taken from two dimensional covalent organic frameworks developed for better heat dissipation (Evans et al., 2021). Usually, silica based low k dielectric materials have a dielectric constant of three or four, better thermal stability and high thermal conductivities. Organic dielectric materials have shown success in obtaining desirable low k dielectric properties even without inducing pores, but only with inadequate heat resistance, mechanical stability and thermal conductivities. Organo-metallic dielectric materials, on the other hand, offer solution but they can be very expensive. Biodielectrics such as bio-inspired peptide nanotubes, for example, have successfully incorporated metal ions with better heat resistance, mechanical stability and thermal conductivities while keeping dielectric constant low. These biodielectrics can be further explored for low k dielectric applications. Porous calcium phosphates such as hydroxyapatites or its derivatives can also be another route to explore.

## 4 Biopiezoelectricity

The breakthrough in the field of dielectrics happened in the year 1880 when French physicists, brothers Jacques and Pierre Curie, discovered piezoelectricity while compressing a certain type of naturally occurring crystals, such as quartz, topaz, Rochelle salt, cane sugar and tourmaline (Koptsik and Rez, 1981). A year later (1881), Lippaman predicted the converse piezoelectric effect, i.e., expansion or contraction of a non-centrosymmetric materials in response to the applied electric field (Tichý et al., 2010), yet the experimental validation was done in the year 2002 (Kornbluh et al., 2002). Piezoelectrics were only of laboratory interest until they were used in sonar applications during World War I by P. Langevin and French co-workers (Szabo, 2004). This led to an increase in research and development of new piezoelectric materials and their applications (King et al., 1990). The piezoelectric effect was later identified and measured in crystals of potassium dihydrogen phosphate (KDP) and ammonium dihydrogen phosphate (ADP) in the early 1940s (Mason, 1946). The ADP crystals were then utilized in high power acoustic transducers (S. Zhang et al., 2015). During the time of World War II, some synthetic piezo-crystals, such as barium titanate (BaTiO_3_ K > 1100) (Vul and Goldman, 1945; Hippel et al., 1946; Iding, 1971), or lead zirconate titanate (PZT) (Cross and Newnham, 1987), were developed and showed relatively high piezoelectric coefficients. The breakthrough in piezoelectric polymers happened in the year 1969 with the discovery of piezoelectricity in poled polymers, such as nylon and polyvinylidene fluoride (PVDF) (Kawai, 1969). Nowadays, PVDF and its copolymers are the most commercially available piezoelectric polymers finding applications in nanogenerators, sensors, energy harvesting, and biomedical fields (Kalimuldina et al., 2020; Xia and Zhang, 2018; Namsheer and Rout, 2021).

Piezoelectricity in biological materials have been widely studied for the last few decades owing to its biocompatibility, natural degradability, environmental friendliness and potential to replace conventional piezoelectrics such as, inorganic/ceramic piezoelectric materials and synthetic polymers in many aspects. A variety of bio-piezoelectric systems have been explored so far. Here, we provide a brief overview of research progress in biological piezoelectric materials precisely amino acids, peptides, and polysaccharide especially cellulose.

Amino acids are essential building blocks for peptides, proteins, and integral components of life. Piezoelectricity in amino acid crystals is the result of non-centrosymmetry in the crystal structure which allows a net polarisation in different directions. In 1970, Vasilescue et al. first experimentally investigated piezoelectricity in amino acid powders (Vasilescu et al., 1970). Since then, a lot of studies have been conducted in proteinogenic amino acid crystals and powders (Lemanov 2000; Guerin et al., 2019). Namely, glycine is a non-chiral amino acid found to exhibit piezoelectricity in its β - glycine and γ - glycine form, which have non-centrosymmetric space group P3_1_ and P3_2_ respectively (Albrecht and Corey, 1939; Guerin et al., 2018a). The highest theoretically obtained shear piezoelectricity (d_16_) value of β glycine is 195 p.m./V, which has been further experimentally established and the measured value is 178 ± 11 p.m./V (Guerin et al., 2018b). In addition, the highest measured longitudinal piezoelectric coefficient (d_33_) value for γ - glycine is 10.4 p.m./V (Kumar et al., 2011). Furthermore, numerous works have been carried out on glycine piezoelectricity till date (Zelenovskiy et al., 2016; Sui et al., 2022; Yang et al., 2021; Zhang et al., 2023a). In addition to glycine crystal, Guerin et al. also explored the piezoelectricity of other amino acid crystals (Guerin et al., 2018; O’Donnell et al., 2020).

Peptides are composed of small chains of amino acids and exhibit piezoelectricity because of the same non-centrosymmetry feature and it includes diphenyl alanine (FF), fluorenylmethyloxycarbonyl diphynylalanine (Fmoc-FF), cyclo-phenylalanine-tryptophan (cyclo-FW), cyclo-glycine-tryptophan (Cyclo-GW) etc (Kholkin et al., 2010; Bosne et al., 2013; Vasilev et al., 2016; Ryan et al., 2015; Tao et al., 2019a; Tao et al., 2019a). Among them, the most investigated natural peptide is FF peptide, and a strong piezoelectric anisotropy was explained from PFM of FF microtubes (d_15_ ∼ 80 ± 15 p.m./V and d_33_ of 18 ± 5 p.m./V) (Vasilev et al., 2016).

In FF-based peptide nanotubes as well as other amino acids and dipeptides, chirality plays an important role in their dielectric properties (Bystrov et al., 2019; Tverdislov et al., 2022) and, in turn, relevant to their bio-ferroelectricity as shown in the works of (Bystrov et al., 2019; Zelenovskiy et al., 2019; Bystrov et al., 2020; Bystrov, 2024). Peptide nanotubes generally exhibit piezoelectric, pyroelectric, and ferroelectric properties. The chirality of the original amino acids and dipeptides changes during their self-assembly into nanotubes, which corresponds to the law of changing the type (sign) of chirality when moving to a higher level of self-organization of molecular structures (Tverdislov et al., 2022). Biomolecular structures in living organisms are built on left-handed amino acids (L-type). Peptide nanotubes based on such left-handed amino acids and dipeptides, in particular, the L-FF are well studied. Recently, Zelenovskiy et al. synthesize peptide nanotubes based on right-handed amino acids (D-type|) and dipeptides (D-FF) (Zelenovskiy et al., 2019). It turned out that in addition to the obvious differences (between these peptide nanotubes, L-FF and D-FF, were different in optical dichroism, obviiuslt but there were also different in mechanical and dielectric properties. For example, peptide nanotubes synthesized based on D-FF templates were shorter and stiffer than those synthesized based on L -FF templates.,Total dipole moment, polarization and piezoelectric coefficients were also different and often 50% greater in D-FF based nanotubes (Zelenovskiy et al., 2019; Bystrov et al., 2020; Bystrov, 2024). Additionally, other self-assembled peptides such as, Fmoc-FF and cyclo-GW displayed the highest piezoelectric co-efficient of d_15_ ∼ 33.7 p.m./V and d_16_ ∼ 14 pC/N (Ryan et al., 2015; Tao et al., 2019b). Peptide nanotubes based on the dipeptides leucine (L) and isoleucine (I) have also been studied in both left-handed (L-LL, L-II) and right-handed chirality (D-LL, D-II) (Bystrov, 2024). Many of the FF nanotubes have been designed for piezoelectric energy harvesting. Proteins are higher ordered structure of peptides composed of multiple amino acids. Piezoelectricity has been found in a variety of proteins such as collagen, elastin, lysozyme, and silk (Yucel, Cebe, and Kaplan, 2011; Harnagea et al., 2010; Denning et al., 2014) etc. Interestingly so many discrepancies can be observed in the experimentally obtained piezo constant values for these protein materials from a limited number of experiments. This inconsistency in measurements for a particular protein material identifies the lack of fundamental understanding about the origin of biopiezoelectricity.

Besides piezoelectricity of amino acids to proteins, plant-based polysaccharide material also exhibits piezoelectric effect. Cellulose is the most abundant polysaccharide, the major component of plant biomass. It’s a fibrous polymeric polysaccharide consisting of β-1,4-linked D-glucose residues. Research activities in cellulose started with studying the piezoelectric effect in wood by Fukada et al. in early 1950s (Fukada, 1955; Fukada, 1968). Since then, a lot of effort has been paid to deconstruct the cellulose piezoelectricity which covers cellulose microfibrils, cellulose films, and cellulose nanocrystals (CNCs) (Rajala, Siponkoski, Sarlin, Mettanen, et al., 2016; Csoka et al., 2012; Zhai et al., 2020). Subsequently, their electromechanical responses have been evaluated both experimentally and theoretically. As an outcome, a wide range of piezoelectric coefficient values (shear, transverse and longitudinal) for different cellulose samples prepared from different sources and preparation methods (Chae et al., 2018). Therefore, it could raise some probes like involvement of other electromechanical coupling during measurements and true origin of the piezoelectricity in these biological materials.In addition, plenty of excellent review papers are reported in last few years explaining the intrinsic piezoelectric properties of different biological materials, preparation methods, piezoelectricity measurement techniques and their application in different fields ranging from mechanical energy harvesting, sensing, tissue regeneration to biomedical engineering (Chae et al., 2018; Wang, Sui, and Wang, 2022; Xu et al., 2021; Kim et al., 2020; Tuszynski, Craddock, and Carpenter, 2008; Amdursky et al., 2010; Li et al., 2013; Bystrov et al., 2014).

## 5 Biopyroelectricity

The effect of pyroelectricity is known for over two millenia since the Greek philosopher Theophrastus wrote, on stones, c. 315 B.C “It [Lyncurium: Tourmaline, found in urine extracts of Lynx] has the power of attraction, just as *amber* has, and some say that it not only attracts straws and bits of woods, but also copper and iron, if the pieces are thin” (Caley and Richards, 1956). Tourmaline is inorganic, trigonal (3m) crystal. Its property of attraction was attributed to electric phenomenon in 1747 by French Physician and Chemist Linné, who called it *lapis electricus. Amber is fossilised tree resin, C*_*12*_ *H*_*20*_ *O having no crystal structure.* “It is asserted, too, that these stones [flame coloured stone known as lychnis], when *heated by the sun* or rubbed between the fingers, will attract chaff and filaments of paper.” Pliny the Elder, *Natural History*, c. First Century, A.D. (Lang, 1974). Lychnis is a kind of ruby (corundum, Al_2_O_3_ trigonal, -3m, with some Cr) “lampstone”. In the year 1824, David Brewster, recognized for his work in optics, was the first author to use the term “pyroelectricity” and to observe pyroelectricity phenomenon in various crystals, one among which was Rochelle salt (Brewster, 1824).

Not long after the discovery of piezoelectricity in bone and tendon, pyroelectricity was found by Lang in 1966 (Lang, 1966) in phalanges and hoof tendons of a cow. Since then, pyroelectricity has been observed in various biological entities which includes amino acids, plant leaves, thorax of live insects, hydroxyapatite, lysozyme, etc (Lang, 1966; Lang and Herbert, 1977; Athenstaedt and Claussen, 1981; Tofail et al., 2009; Lang et al., 2011; Stapleton et al., 2018; Lemanov 2000). It was previously concluded that the pyroelectric effect of bone originated from collagen but not from hydroxyapatite based on the formerly established centrosymmetric crystallographic space group for hydroxyapatite, which precluded piezoelectricity. Later on, the crystal group assignment was corrected, which allows both piezo- and pyroelectricity. The measured pyroelectric co-efficient for unpoled hydroxyapatite ceramics is 12 μC/m^2^K, whereas that of poled hydroxyapatite is ranging from 0.1 to 40 nC/cm^2^ K at 300°C–500 °C (Tofail et al., 2009; Lang et al., 2011).

In addition, Martin first observed pyroelectric and piezoelectric phenomena in bundles of wool and hair (Martin, 1941). Athenstaedt investigated pyroelectricity in wheat and showed the temperature dependence of the pyroelectric constant for the epidermis of wheat grains (Athenstaedt, 1976). It is interesting to note that winter wheat and spring wheat show quite different results, reflecting the different temperature variation of residual polarisation in the grains. It is assumed that the polarisation in winter wheat decreases more sharply than that in spring wheat with increasing temperature.

Fukada et al. found that thin films of aromatic polyurea shows pyroelectricity. pyroelectric activity is generated in these films by a poling treatment (Takahashi et al., 1989)]. The orientation of urea bonds (NH- CO-NH) with a dipole moment of 4.9 D is responsible for a large residual polarisation in these films.

The pyroelectric coefficient reported for γ glycine (13 × 10^−6^ C/m^2^K) is more than three times higher than that of tourmaline, illustrating that strong pyroelectricity is present even in the smallest biological building blocks (Lemanov 2000). Pyroelectric properties of some glycine-based materials are even higher.

In 2018, Stapleton et al. investigated the pyroelectricity in polycrystalline aggregate films of globular protein lysozyme prepared on IDE electrode and the measured pyroelectric coefficient is 1441 ± 536 μC/m^2^K (Stapleton et al., 2018). The coefficient is the till date highest obtained among all biological materials and comparable to PZT grown on SrTiO_3_ substrate (Botea et al., 2013). In addition, Kholkin et al. discovered pyroelectric effect and polarisation instability after a certain temperature change in self-assembled diphenyl alanine microtubes. The obtained pyroelectric co-efficient for the micro tube bundle is 2 μC/m^2^K (Esin et al., 2016). Interestingly, a paper by Tofail et al. listed and compared the pyroelectric coefficients of different biological materials (Tofail, 2023).Recently, Kim et al. demonstrated heat induced electrical polarisation in virus. Precisely, they investigated the pyroelectric properties of vertically aligned M13 bacteriophage film and obtained a pyroelectric coefficient of 0.13 μC/m^2^ °C (Kim et al., 2023). It may pave the way for new bio-inspired pyroelectric devices.

## 6 Bioferroelectricity

As it has been discussed in previous sections, a subgroup of polar dielectrics, i.e., piezoelectric and pyroelectric materials can be ferroelectric exhibiting hysteresis, polarisation switching, and Curie point below or beyond which ferroelectric state may cease to exist. Hysteresis in ferroelectric materials occurs through interaction between the electric field and the material, which is polar. In linear or paraelectric dielectric, polarisation returns to its original unpolarised, “virgin” state when the applied electrical field is removed. Ferroelectrics retain their polarisation after the field is removed; the amount of this polarisation is known as Remnant polarisation (P_R_). The state of zero polarisation can be restored by applying an electrical field in the direction that is opposite to the direction of the original applied field. This switching field is the electric coercivity (E_C_). The maximum amount of polarisation that can be induced in the material at high electric field strength is known as the saturation polarisation (P_s_).

An electret, again, derived from drawing analogy with a magnet, retains electrical charge and/polarisation quasi-permanently. Electrically charged and/or polarised state can be naturally present or induced by charge injection to create space charge or dipolar orientation/reorientation. Ferroelectrets, in practice, generally includes space-charge electret foams and films with cavities that, when charged, form macroscopic dipoles that can be switched by reversing the polarity of the charging electrical field. These materials show hysteresis like traditional ferroelectrics as well as piezo and pyroelectric behaviours and can be useful as nonlinear components in many systems and circuits, memory devices as well as energy generation, sensing and actuation. Unlike ferromagnetic materials, ferroelectrics have only a limited level of adoption. Newer areas of electro-optics and electronics can take advantage of ferroelectrics to create a broad range of devices, however.

Since the discovery of piezoelectric and pyroelectric properties in bone in the 1950s and 1960s respectively, questions arose about ferroelectricity in biological systems. The focus of the curiosity was mainly on the anatomical structure, which, in the case of bone was, initially attributed to its mineral constituent, hydroxyapatite (Shamos and Lavine, 1967). A. R. von Hippel suggested in 1970 that true relations might exist between ferroelectricity, liquid crystals formation, and the generation of electric impulse in nerves and muscles (Hippel, 1970). Experimental evidence of ferroelectricity alluded anticipation, however, as ferroelectric hysteresis in the classical sense was investigated in biomass and its building blocks. Ferroelectric behaviour as neat as those observed in non-biological systems has rarely been observed. Biological systems contain water, a polar molecule with relatively high dielectric constant (∼80 in the free state). The presence of water is deleterious in classical measurements of ferroelectricity due to charge leakage. Biological systems also contain bound water, to complicate matters further. Kubisz et al. (1984) investigated collagen from Achilles tendon with a 10% water content for a hypothetical dielectric hysteresis loop that showed a maximum shift along the direction of the electric field applied in the vicinity of 375 K. However, very large dielectric losses at low frequencies made it difficult to interpret the collagen ferroelectricity.

The measurement of an electrical field dependent polarisation-hysteresis and promptly jumping into claim of ferroelectricity has remained a practice for quite some time. For example, Lemnaov et al. questioned the experimental data presented in the 1960s when claiming ferroelectricity in deoxyribonucleic acid (DNA) and ribonucleic acid (RNA). On the other hand, there are more careful and skeptical observations of hysteresis that have often been overlooked in the discussion of ferroelectricity in biological systems. For example, a plot of the polarisation versus the applied electric field revealed an apparent ferroelectric character in single crystal chlorapatite, a chlorine substitute of mineral analogue of bone mineral hydroxyapatite. The authors did not claim ferroelectricity as such a claim would have been in conflict with their original crystallographic conviction that apatite should have belong to nonpolar space groups such as P63/m or P21/b. Haverty et al. provided evidence that polar space groups are indeed possible for apatite, yet hydroxyapatite should not be ferroelectric due to crystallographic restrictions. They corrected this promptly by using a ferroelectric to antiferroelectric transition in hydroxyapatite to explain an experimentally observed dielectric anomaly in hydroxyapatite ceramics. The claim of ferroelectricity in hydroxyapatite was then substantiated by contact poling and e beam poling of hydroxyapatite ceramics and thin films and measurement of hysteresis in poled hydroxyapatite. Hydroxyapatite can be in both ferroelectric and non-ferroelectric states depending on the orientation of its hydroxyl (OH) groups within the OH channel of the hydroxyapatite crystal structure (Bystrov, 2015; Bystrov, 2019; Hu et al., 2017; Lang, 2016). This was clearly seen both experimentally (Lang, 2016) and theoretically from atomistic simulations (Bystrov, 2015; 2019; Hu et al., 2017). Physiological impact of such polarisation switching was demonstrated by showing selective attachment of lysozyme, an enzyme that has an electropositive charge envelop in natural pH of body fluid. The group then went on to discover piezo, pyro and ferroelectricity in lysozyme crystal. Ferroelectricity in amino acids glycine and thymine have also been reported (Bystrov et al., 2015).

Turning to the synthetic constructs of biological building blocks provided a much better platform to measure properties of biodielectrics and critically reevaluate historic claims of ferro-, pyro- and piezo-electricity in hierarchical structures. Additionally, the approach has opened the probability of finding technologically relevant biological materials as well as physiological relevance of dielectric properties of biological building blocks and their hierarchical structural organisations. Importantly, the approach can provide a new premises where parallel developments in the field of electrophysiology can actually converge with the observation of dielectric properties of biological structures. For example, when Cole and Curtis first demonstrated that the action potential was associated with a large increase in the conductance of the cell membrane (Cole and Howard, 1939), we understood that this behaviour is similar to a leaky capacitor between ion-conducting intra and extracellular fluids. Hodgkin and Huxley provided fundamental insights into nerve cell excitability that led to the understanding of voltage-gated ion channels (VGICs) give rise to propagating action potentials, but also the very framework for studying and analysing ion channel kinetics (Hodgkin and Huxley, 1939; Hodgkin and Huxley, 1952). The magnitude of piezoelectricity of collagen can provide such voltage under stress in physiological conditions (Guerin et al., 2023).

In 1992, Beresnev et al. (1992) alluded a similarity between biological membranes built of multilayer structure with tilted lipid and protein molecules and ferroelectric liquid crystals (Goodby, 1991). Ferroelectricity was considered to be an underlying mechanism behind the propagation of excitation in biological membranes. Earlier on, Leuchtag (Leuchtag, 1987a; Leuchtag, 1987b; Leuchtag, 1995) proposed ferroelectric models of ion channels of the excitable biological membranes, where the opening/closing processes of these channels ensure the passage of ion currents (Na/K) resulting in membrane’s resting potential and action potential in accordance of a ferroelectric phase transition. The nonlinear behavior of dielectric permittivity k in the vicinity of this phase transition depended on temperature and electric field. This was an important difference from the Hodgkin-Huxley model (Hodgkin and Huxley, 1952), in which the dielectric permittivity k was independent of temperature and electric field. In fact, Leuchtag observed (Leuchtag, 1995) the existence of Curie-Weiss law (Lines and Glass, 2001) of ferroelectric transition, through the nonlinear behavior of the dielectric permittivity k in the ion channels of an excitable biological membrane. Experimental data of the temperature variation of capacitance of a biological membrane consisting of ion channels (Palti and Adelman, 1969) exhibited ferroelectric nature (Lines and Glass, 2001). Leuchtag, Bystrov and others continued this line of enquiry (Bystrov, 1992; Bystrov and Leuchtag, 1994; Bystrov et al., 1994; Leuchtag and Bystrov, 1999) that paved the way to finding bio-ferroelectricity and bio-piezoelectricity in bio-molecular structures such as peptide nanotubes (Bystrov et al., 2019; Tverdislov et al., 2022; Zelenovskiy et al., 2019; Bystrov et al., 2020; Bystrov, 2024), which are discussed previously later in this article. The role of internal electric fields in living systems, were also analyzed in detail by Leuchtag’s in his book (Leuchtag, 2008), including ferroelectric phenomena (Lines and Glass, 2001; Goodby, 1991) associated with electric fields in various biomolecular systems.

In terms to physiological relevance of ferroelectricity, Leuchtag and Bystrov (Leuchtag and Bystrov, 1999) suspected two distinct types of biological structures to have ferroelectric properties: microtubules and voltage-dependent ion channels found widely available in cell membranes that regulates bioelectric signal conduction in nerve and muscle cells. Both microtubules and ion channels involve proteins with the possibility of information processing at the cellular level. Leuchtag and Bystrov (Leuchtag and Bystrov, 1999), outlined the principles of bioferroelectricity defining the field as one dealing with ferroelectricity and related phenomena in biological systems. Important work on microtubules, and the passage of action potentials and nerve impulses were considered where ferroelectricity models can be applied (Brown and Tuszynski, 1999; Gordon et al., 1999; Tasaki, 1999). Leuchtag (Leuchtag, 2023), has recently elaborated a condensed matter approach to link ferroelectricity with the operation of ion channels of excitable biological membranes.

Proton pumping membrane protein cytochrome C ba3 oxidase has been found to exhibit out of plane and in plane piezoelectricity when measured in piezo response force microscopy suggesting a pyroelectricity is possible. Ferroelectricity has not been reported. Fibrillar proteins such as collagen have been reported to be both ferro and antiferroelectric. Elastin, another highly explored protein-based macromolecule in organs such as the skin, blood vessel walls, and the lungs of the human body, has also been reported to be piezoelectric (Fukada and Hara, 1969) and ferroelectric (Liu et al., 2014). The claim of ferroelectricity in aortic vessel wall made of muscle protein elastin was later refuted from conventional Sawyer- Tower measurements.

## 7 Application of biodielectric materials

Owing to the biocompatibility and biodegradability of bio-dielectric materials, they are the promising candidates for different applications such as biomechanical energy harvesting, tissue engineering, sensing and precisely for implantable biomedical applications. However, it is too early to claim the competency of bio-dielectrics compared to the well-established synthetic polymers and ceramic dielectric materials. In this section, we will demonstrate a brief idea about the ongoing research progress towards different directional applications of bio-dielectrics.

Biomechanical energy harvesting especially focuses on collecting and converting the mechanical energy associated to different motions like joint movement, muscle bending-stretching, heartbeat and breathing (Roy et al., 2021; Roy et al., 2020)] through piezoelectric phenomenon. Additionally, the time dependent temperature fluctuation during inhale-exhale process can also be used for pyroelectric energy harvesting (Ghosh et al., 2020; Roy et al., 2019). Among the bio-piezoelectric materials, cellulose, silk, collagen, lysozyme, viruses, amino acids and peptide-based materials have been widely cultivated for piezoelectric device, i.e., nanogenerator (NG) fabrication and used as implantable devices, motion sensors, human physiological signal monitoring and charging portable devices (Rajala et al., 2016; Yun et al., 2008; Maiti et al., 2017; Ghosh et al., 2017; Karan et al., 2018; Yucel et al., 2011; Lee et al., 2018; Nguyen et al., 2016; Yang et al., 2021; Zhang et al., 2023b; Ghosh and Mandal, 2016b; Ghosh and Mandal, 2016a; Roy et al., 2024). In addition, bio-inspired (i.e., composite biomaterials) systems have been designed for piezoelectric devices to harvest energy from different body motions and to improve the piezo-output (Vivekananthan et al., 2018; Maity et al., 2018; Zheng et al., 2016; Wu et al., 2021). Therefore, bio-piezoelectric devices can serve the purpose of self-powered pressure or force sensors. The most promising parts of using these materials are their natural abundance and complete degradation in body environment without causing any adverse effect. Still there remains some limitations which need to be improved and established to identify an economical way to construct a bio-piezoelectric device, for example, low piezoelectric co-efficient and energy conversion efficiency, difficulty in large scale assembly, lack of understanding of the origin of piezoelectricity and surface charge contributions during measurements.

Another interesting side of bio-dielectric based application is to develop tissue scaffolds for tissue regeneration. After the discovery of bone piezoelectricity, bio-piezoelectric materials particularly collagen and chitosan have been intensively studied to construct biomimetic tissue scaffolds to facilitate tissue growth and bone regeneration using electrical stimulation (ES) (Chen et al., 2019; Chen et al., 2020b; Prokhorov et al., 2020; Ribeiro et al., 2015; Goonoo and Bhaw-Luximon, 2022; Zaborowska et al., 2010). Fernandez-yague et al. demonstrated the effect of collagen analogue based bio-piezoelectric device on the dynamic response of tendon cells in a rat achilles acute injury model by modulating the response of ion channels and specific tissue regeneration signalling pathways (Fernandez-Yague et al., 2021). Moreover, Du et al. fabricated a PEDOT/chitosan core shell nanofibers and showed the synergetic interplay between external ES and piezoelectric voltage for nerve tissue engineering (Du et al., 2020). Therefore, it is obvious that the ES-guided tissue engineering has promising advantages in the field of postinjury tendon regeneration, self-powered healing and bone regeneration.

## 8 Conclusion

The field of biodielectrics is old but has attracted new interests in both fundamental science and technological applications. In this article, we have discussed these renewed interests in the field within the purview of its historical context. We have provided a critical discussions on the developments in the field of piezo, pyro, and ferroelectricity in biological and bioinspired materials as well as potential for such materials in, for example, low k dielectric applications due to their inherently low permittivity. We conclude that biodielectrics will play an important role in our pursuit of developing sustainable materials for next-generation technologies.
